# Oxidative Metabolism Genes Are Not Responsive to Oxidative Stress in Rodent Beta Cell Lines

**DOI:** 10.1155/2012/793783

**Published:** 2012-02-20

**Authors:** Faer Morrison, Karen Johnstone, Anna Murray, Jonathan Locke, Lorna W. Harries

**Affiliations:** Institute of Biomedical and Clinical Sciences, Peninsula College of Medicine and Dentistry, University of Exeter, Barrack Road, Exeter EX2 5DW, UK

## Abstract

Altered expression of oxidative metabolism genes has been described in the skeletal muscle of individuals with type 2 diabetes. Pancreatic beta cells contain low levels of antioxidant enzymes and are particularly susceptible to oxidative stress. In this study, we explored the effect of hyperglycemia-induced oxidative stress on a panel of oxidative metabolism genes in a rodent beta cell line. We exposed INS-1 rodent beta cells to low (5.6 mmol/L), ambient (11 mmol/L), and high (28 mmol/L) glucose conditions for 48 hours. Increases in oxidative stress were measured using the fluorescent probe dihydrorhodamine 123. We then measured the expression levels of a panel of 90 oxidative metabolism genes by real-time PCR. Elevated reactive oxygen species (ROS) production was evident in INS-1 cells after 48 hours (*P* < 0.05). TLDA analysis revealed a significant (*P* < 0.05) upregulation of 16 of the 90 genes under hyperglycemic conditions, although these expression differences did not reflect differences in ROS. We conclude that although altered glycemia may influence the expression of some oxidative metabolism genes, this effect is probably not mediated by increased ROS production. The alterations to the expression of oxidative metabolism genes previously observed in human diabetic skeletal muscle do not appear to be mirrored in rodent pancreatic beta cells.

## 1. Introduction

Type 2 diabetes (T2D) occurs when the pancreatic beta cells can no longer compensate for peripheral insulin resistance by increasing insulin production and is associated with hyperglycemia and an altered lipid profile (dyslipidemia) [1]. The T2D “microenvironment” is detrimental to cells and tissues and is thought to contribute to further beta cell dysfunction and reduced beta cell mass, as well as microvascular and macrovascular complications. Increased levels of reactive oxygen species (ROS) are hypothesized to have a role in causing beta cell dysfunction due to altered glucose levels and lipid profiles, leading to T2D [2]. ROS have a physiological role in normal intracellular signal transduction. However, excessive ROS production causes damage to cellular components, of which RNA is especially vulnerable, potentially leading to gene expression changes [3].

Mitochondrial dysfunction is thought to contribute to beta cell dysfunction and has been observed in beta cells and in other tissues of individuals with T2D [4, 5]. Moreover, several groups have found that components of the electron transport chain and other genes involved in oxidative metabolism were altered in tissues from individuals with T2D [6–8]. For example, decreases in the expression of oxidative phosphorylation genes regulated by the transcriptional coactivator PGC1 (PPARGC1A), which is involved in regulation of energy metabolism, have been observed in T2D skeletal muscle [6, 7]. Increases in the expression of these genes were observed in liver from patients with T2D and were correlated with blood glucose levels [8].

The beta cells of the pancreas are particularly vulnerable to the effects of ROS as they contain lower levels of antioxidant enzymes (catalase, superoxide dismutase, glutathione peroxidase) compared with other tissues, including skeletal muscle and liver [9]. ROS-mediated mitochondrial dysfunction, as observed in T2D islets, has been shown to disrupt glucose-induced insulin secretion from beta cells [10]. Therefore, the beta cells, as well as being the central tissue in T2D pathogenesis, might be expected to be particularly susceptible to ROS-mediated gene expression changes.

The vulnerable state of the beta cell and the importance of oxidative metabolism in relation to insulin secretion and ROS production led us to investigate whether the expression of genes involved in oxidative metabolism is altered in response to hyperglycemia-induced oxidative stress in a rodent pancreatic beta cell line INS-1. We hypothesized that hyperglycemia-induced oxidative stress in the pancreatic beta cell may contribute to beta cell dysfunction and impaired insulin secretion because of deregulation in the expression of oxidative metabolism genes.

## 2. Materials and Methods

### 2.1. Cell Culture and Experimental Procedure

The rat pancreatic beta cell line INS-1 was cultured in RPMI 1640 medium (Invitrogen) supplemented with 11 mmol/L glucose, 10% fetal calf serum and 1% penicillin/streptomycin at 37°C in a humidified atmosphere. After 72 hours, the cells were seeded in 25 cm^2^ flasks at a density of 3.5 × 10^5^ cells/flask (for gene expression analysis) or in 96-well plates at a density of 2 × 10^4^ cells/well (for cell viability and ROS production measurements). Cells were then incubated under the conditions already described but with low (5.6 mmol/L), ambient (11 mmol/L) or high (28 mmol/L) glucose for a further 48 hours (conditions previously used to model hyperglycemia in T2D) [11].

### 2.2. Cell Viability

Cell viability was measured using (3-(4,5-dimethylthiazol-2-yl)-2,5-diphenyltetrazolium bromide (MTT) assay [12]. Briefly, MTT was added to cells at a final concentration of 0.5 mg/mL. Cells were incubated for 1 hour at 37°C. The medium was then aspirated and 100 *μ*L DMSO added to each well to solubilize the blue formazan product. Absorbance was measured using an OPTIMA reader (BMG LABTECH) at an excitation wavelength of 540 nm. Three biological replicates were carried out, each with five technical replicates.

### 2.3. ROS Production

Intracellular ROS production was measured using the fluorescent probe dihydrorhodamine 123 (DHR123) (Invitrogen), which, when oxidized, localizes in the mitochondria and fluoresces green, indicating the presence of ROS. In brief, DHR was added to cells in 100 *μ*L fresh RPMI medium to a final concentration of 1 *μ*M. Cells were incubated for 30 minutes at 37°C. Fluorescence was measured using a PHERAstar reader (BMG LABTECH) at an excitation wavelength of 485 nm and an emission wavelength of 520 nm. Three biological replicates were carried out, each with five technical replicates.

### 2.4. Gene Expression Analysis

The genes selected for this study are given in Table 1. Choice of targets was made on the basis that these 90 genes have shown evidence in the literature that they may be effected by some of the physiological changes that occur with T2D. The first set of targets (indicated in Table 1 by bold type) were taken from a study where microarray analysis of skeletal muscle samples from matched diabetic and nondiabetic subjects was undertaken [7]. Using a pathways analysis approach, they identified a set of genes involved in oxidative phosphorylation whose expression was decreased in diabetic muscle. The majority of these targets were genes responsive to the transcriptional coactivator PGC1. We therefore chose to study these, together with other PPAR genes (*Ppara*, *Ppard,* and *Pparg*) and their targets. This is relevant because the Pro12Ala variant of PPARG has been associated with T2D [13]. A very similar pattern of gene expression was also noted by a second group, who carried out an analogous experiment, also in skeletal muscle [6]. In concordance with the Mootha study, this study demonstrated deregulation of a group of genes involved in oxidative phosphorylation regulated by nuclear respiratory factor-1 (NRF1) and PGC1 (indicated in Table 1 by underlined type). Genes that appear in both studies are marked in Table 1 by bold and underlined type. Other targets have been selected on the basis of involvement in response to oxidative stress and with roles in oxidative metabolism. Most of these genes can be subdivided into activation of the antioxidant defense system, cell cycle arrest, DNA repair, damaged protein repair, or activation of the NF*κ*B pathway. The final category of genes were selected on the basis that they are key players in pathways involved in T2D. These include genes involved in cell cycle and apoptosis, immune and inflammatory processes, energy metabolism and homeostasis, including glucose metabolism, and insulin signaling and homeostasis. Expression of the 90 target genes were analyzed with the Micro Fluidic Card system (Taqman low density array (TLDA) custom array, Applied Biosystems).

### 2.5. Statistical Analysis

Comparisons of ROS production and gene expression between the three glucose culture conditions were determined using the Kruskal-Wallis *H* test.

## 3. Results and Discussion

**Figure 1:**
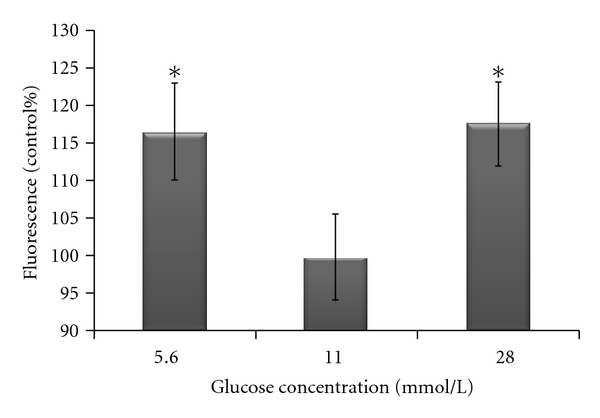
The production of ROS in INS-1 cells cultured in low, ambient, and high glucose concentrations for 48 hours. Intracellular ROS production was measured using the fluorogenic probe dihydrorhodamine 123. Differences in ROS production were statistically analyzed by the Kruskal-Wallis *H* test. Significant results (*P* < 0.05) relative to control are indicated by*.

**Figure 2:**
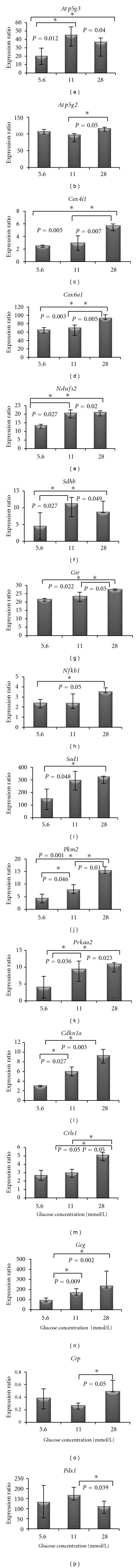
Gene expression in INS-1 cells cultured in low (5.6 mmol/L), ambient (11 mmol/L), and high (28 mmol/L) glucose concentrations for 48 hours (gene name above each graph). Gene expression changes were analyzed using TLDA expression profiling on a panel of 90 target genes. Gene expression differences were statistically analyzed by the Kruskal-Wallis *H* test and were normalized to the mean expression of the endogenous controls *B2m* and *Tbp, *as they were found to be most stable using the GeNorm algorithm (Statminer, Integromics). Note that the scales of the graphs differ between genes as the expression is shown relative to mean expression across all genes. Significant results (*P* < 0.05) are indicated by*.

We found that although ROS were increased at both high and low glucose compared with ambient glucose (*P* < 0.05) (Figure 1), this was not accompanied by concomitant alterations in the expression levels of the 90 test genes. TLDA analysis revealed deregulation of 16 out of 90 (18%) of genes analyzed (*P* < 0.05) (Figure 2), but the patterns of deregulation did not mirror changes in ROS production. If increased ROS production was responsible for the changes in gene expression, then we would expect the pattern of ROS production to mirror the pattern of gene expression. This, however, was not the case, which leads us to conclude that the expression changes are probably due to effects of glycemia, rather than a specific effect of ROS. The lack of response in the remaining 82% of genes tested may indicate that these genes are not responsive to ROS or glucose in beta cells. A decrease in cell viability was observed at low glucose after 48 hours, whereas high glucose increased cell viability compared with ambient glucose (results not shown). The effects on cell viability could explain some of the gene expression changes observed.

This study provides evidence that increasing glycemia affects expression of a proportion of genes involved in oxidative metabolism in the pancreatic beta cell line INS-1. A number of genes were upregulated in response to increasing glycemia, including several components of the electron transport chain (*Atp5g3, Atp5g2, Cox4i1, Cox6a1, Ndufs2, Sdhb*) (Figures 2(a)–2(f)) and genes involved in oxidative metabolism or cellular antioxidant defense (*Gsr, Nfkb1, Sod1*). Also upregulated with high glucose were genes involved in energy homeostasis or metabolism (*Pkm2, Prkaa2*) (Figures 2(g)–2(k)). Deregulation of these genes in beta cells could potentially contribute to impaired mitochondrial metabolism and insulin secretion. *Prkaa2* is a catalytic subunit of the AMP-activated protein kinase (AMPK), a key regulator of energy homeostasis, which has been shown to decrease glucose-stimulated insulin secretion, insulin content, and mitochondrial metabolism [14].

Also up-regulated in response to increasing glycemia are *Cdkn1a* which is involved in p53-mediated cell cycle arrest in response to cellular stress and has already been shown to be induced by H_2_O_2_ [15], *Crls1* which is important in maintaining the integrity of the mitochondrial membrane, and is thought to be involved in apoptosis, *Gcg* which encodes four distinct proteins including glucagon, and the inflammatory marker *Crp* (Figures 2(l)–2(o)). Interestingly, the only gene that is significantly downregulated in response to high glucose, compared with ambient glucose, is the promoter of beta cell function and survival, *Pdx1 *(Figure 2(p)). It has been shown previously that *Pdx1* deficiency causes beta cell dysfunction and beta cell death and that both hyperglycemia and hyperlipidemia lead to decreased *Pdx1* expression and consequent beta cell dysfunction [16]. The glycolysis gene *Pkm2* is worth mentioning in more detail, as it was the only gene which was significantly deregulated between low and ambient glucose, ambient and high glucose, and low and high glucose (Figure 2(j)). *Pkm2* has previously been shown to be glucose-responsive so acts here as a positive control [17].

Although we saw evidence of deregulated gene expression in response to altered glycemia, we found little evidence to suggest that ROS were involved in mediating these gene expression changes. This is interesting because ROS production and oxidative stress have been strongly associated with mitochondrial dysfunction, and H_2_O_2_-induced oxidative stress has previously been shown to alter expression of some of these genes in beta cells [15, 18]. For instance, H_2_O_2_-induced oxidative stress in rat pancreatic islet cells has been demonstrated to induce *Cdkn1a* mRNA expression [15]. Elevated *Cdkn1a* expression may result in a suppression of beta cell proliferation and insulin biosynthesis, which provides an important link between oxidative stress and beta cell dysfunction in T2D [15]. A transient exposure of the rat insulinoma cell line INS-1E to H_2_O_2_ significantly increased mitochondrial ROS production and impaired glucose-stimulated insulin secretion, which persisted for several days after the exposure [18]. This occurred alongside a concomitant decrease in expression of genes involved in mitochondrial biogenesis and a compensatory increase in expression of respiratory chain subunit mRNAs [18].

Moreover, there are examples of ROS altering signaling pathways in other tissues which are relevant to diabetes, such as adipose tissue and liver. For instance, *in vivo* exposure to high glucose in rats increased the mRNA levels of several inflammatory genes in the adipose, and this effect was partially prevented by the free radical scavenger N-acetyl-cysteine [19]. The authors suggest that exposure to ROS-induced damage over the lifetime of an adipocyte could contribute to the pathological state seen in metabolic disorders such as T2D [19]. ROS levels have been shown to be elevated in the liver of *db/db* mice and in a human hepatic cell line treated with the fatty acid palmitate. The NADPH oxidase NOX3 was found to be the predominant source of ROS production, and the increase in ROS was found to induce p38MAPK and JNK pathways, which was shown to contribute to hepatic insulin resistance [20].

Our panel of 90 genes were selected on the basis that they have already been shown to be deregulated in T2D tissues or are involved in the oxidative stress response, therefore, were strong candidates for this study. Although ROS appear to have little role in mediating the expression of these genes under the conditions described, it should be highlighted that ROS may influence the expression of other genes involved in beta cell function. It would be interesting to further investigate the effect of hyperglycemia-induced ROS production on expression of other genes with important roles in beta cell function, such as maintenance of beta cell mass and regulation of apoptosis, as both hyperglycemia and oxidative stress are thought to be crucial in mediating beta cell apoptosis and subsequent loss of beta cell mass in T2D [21].

In conclusion, our study provides further evidence that hyperglycemia induces ROS production in the pancreatic beta cell. Although 18% of the oxidative metabolism genes tested were shown to be deregulated in response to increasing glycemia, there was little evidence that ROS or oxidative stress was involved in mediating these gene expression changes, indicating that gene expression changes noted in diabetic tissues may be more attributable to differences in glucose or lipid concentration than to increases in oxidative stress.

## Figures and Tables

**Table 1:** Panel of 90 target genes for analysis by TLDA expression profiling. Candidate genes have been shown by pathway-based microanalysis to be deregulated in T2D skeletal muscle, are reported in the literature to be involved in oxidative metabolism or the oxidative stress response, or are reported in the literature to be key players in diabetes pathways/deregulated in T2D.

Panel of 90 target genes for analysis by TLDA expression profiling							
Deregulated in T2D skeletal muscle	***Alas1 ***	***Atp5c1 ***	***Atp5g1 ***	***Atp5g2 ***	***Atp5g3 ***	***Ckmt1 ***	***Cox4i1 ***
	***Cox6a1 ***	***Cox6c ***	***Nrf1 ***	***Pdx1 ***	***Pkm2 ***	***Por ***	***Sdhc ***
	***Slc25a4 ***	***Ucp2 ***	***Uros ***	***Eif2ak3 ***	***Sdhb ***	***Uqcrc1 ***	***Cox5b ***
	*Atp5o*	*Cox7b *	*Cyc1 *	*Atp5j *	*Ndufa5 *	*Ndufa8 *	*Ndufb5 *
	*Ndufb6 *	*Ndufs2 *	*Ndufs3 *	*Ppara *	*Ppard *	*Pparg *	*Ppargc1a *
	*Sdha *						

Involved in oxidative metabolism and the oxidative stress response	*Aco1 *	*Ccng1 *	*Cdkn1a *	*Cxcl10 *	*Ddit3 *	*Dnaja1 *	*Fmo1 *
	*Gck *	*Gsr *	*Hspa4 *	*Hspa5 *	*Igfbp2 *	*Il18 *	*Ins1 *
	*Ireb2 *	*Nfkb1 *	*Nfkbia *	*Pck1 *	*Rad23a *	*Sod1 *	*Tnf *
	*Tp53 *	*Txn2 *	*Txnip *	*Ung *	*Xbp1 *	*Ercc1*	*Mtor*
	*Nampt*	*Ndufaf1*	*Nfe2l2*	*Prkcd*	*Prkcz*	*Shc1*	

Key players in T2D pathways							

Cell cycle and apoptosis	*Crls1*	*Hdac4*	*Hdac5*	*Hgf*	*Igf1*	*Suv39h1*	
Cellular and energy metabolism and homeostasis, including glucose homeostasis	*Foxo1*	*Gckr *	*Hagh*	*Hk1*	*Prkaa2*	*Sirt1*	
Immune and inflammatory processes	*Crp*	*Il1b*	*Il1rn*	*Il6*	*Rage*		
Insulin signaling and homeostasis	*Gcg*	*Insr*	*Kcnj11*				

## References

[1] Cnop M (2008). Fatty acids and glucolipotoxicity in the pathogenesis of Type 2 diabetes. *Biochemical Society Transactions*.

[2] Robertson AP (2004). Chronic oxidative stress as a central mechanism for glucose toxicity in pancreatic islet beta cells in diabetes. *Journal of Biological Chemistry*.

[3] Kong Q, Lin CLG (2010). Oxidative damage to RNA: mechanisms, consequences, and diseases. *Cellular and Molecular Life Sciences*.

[4] Anello M, Lupi R, Spampinato D (2005). Functional and morphological alterations of mitochondria in pancreatic beta cells from type 2 diabetic patients. *Diabetologia*.

[5] Kelley DE, He J, Menshikova EV, Ritov VB (2002). Dysfunction of mitochondria in human skeletal muscle in type 2 diabetes. *Diabetes*.

[6] Patti ME, Butte AJ, Crunkhorn S (2003). Coordinated reduction of genes of oxidative metabolism in humans with insulin resistance and diabetes: potential role of PGC1 and NRF1. *Proceedings of the National Academy of Sciences of the United States of America*.

[7] Mootha VK, Lindgren CM, Eriksson KF (2003). PGC-1*α*-responsive genes involved in oxidative phosphorylation are coordinately downregulated in human diabetes. *Nature Genetics*.

[8] Misu H, Takamura T, Matsuzawa N (2007). Genes involved in oxidative phosphorylation are coordinately upregulated with fasting hyperglycaemia in livers of patients with type 2 diabetes. *Diabetologia*.

[9] Lenzen S (2008). Oxidative stress: the vulnerable *β*-cell. *Biochemical Society Transactions*.

[10] Sakai K, Matsumoto K, Nishikawa T (2003). Mitochondrial reactive oxygen species reduce insulin secretion by pancreatic *β*-cells. *Biochemical and Biophysical Research Communications*.

[11] Cunha DA, Hekerman P, Ladrière L (2008). Initiation and execution of lipotoxic ER stress in pancreatic *β*-cells. *Journal of Cell Science*.

[12] Mosmann T (1983). Rapid colorimetric assay for cellular growth and survival: application to proliferation and cytotoxicity assays. *Journal of Immunological Methods*.

[13] Florez JC, Hirschhorn J, Altshuler D (2003). The inherited basis of diabetes mellitus: implications for the genetic analysis of complex traits. *Annual Review of Genomics and Human Genetics*.

[14] Rutter GA, Da Silva Xavier G, Leclerc I (2003). Roles of 5′-AMP-activated protein kinase (AMPK) in mammalian glucose homoeostasis. *Biochemical Journal*.

[15] Kaneto H, Kajimoto Y, Fujitani Y (1999). Oxidative stress induces p21 expression in pancreatic islet cells: possible implication in beta-cell dysfunction. *Diabetologia*.

[16] Fujimoto K, Polonsky KS (2009). Pdx1 and other factors that regulate pancreatic *β*-cell survival. *Diabetes, Obesity and Metabolism*.

[17] Yamada K, Noguchi T (1999). Nutrient and hormonal regulation of pyruvate kinase gene expression. *Biochemical Journal*.

[18] Li N, Brun T, Cnop M, Cunha DA, Eizirik DL, Maechler P (2009). Transient oxidative stress damages mitochondrial machinery inducing persistent *β*-cell dysfunction. *Journal of Biological Chemistry*.

[19] Lin Y, Berg AH, Iyengar P (2005). The hyperglycemia-induced inflammatory response in adipocytes: the role of reactive oxygen species. *Journal of Biological Chemistry*.

[20] Gao D, Nong S, Huang X (2010). The effects of palmitate on hepatic insulin resistance are mediated by NADPH oxidase 3-derived reactive oxygen species through JNK and p38 MAPK pathways. *Journal of Biological Chemistry*.

[21] Lupi R, Del Prato S (2008). *β*-cell apoptosis in type 2 diabetes: quantitative and functional consequences. *Diabetes and Metabolism*.

